# Experimental Comparison of Primary and hiPS-Based In Vitro Blood–Brain Barrier Models for Pharmacological Research

**DOI:** 10.3390/pharmaceutics14040737

**Published:** 2022-03-29

**Authors:** Karin Danz, Tara Höcherl, Sascha Lars Wien, Lena Wien, Hagen von Briesen, Sylvia Wagner

**Affiliations:** Fraunhofer Institute for Biomedical Engineering IBMT, Joseph-von-Fraunhofer-Weg 1, 66280 Sulzbach, Germany; karin.danz@ibmt.fraunhofer.de (K.D.); tara.hoecherl@web.de (T.H.); sascha.wien@ibmt.fraunhofer.de (S.L.W.); lena.wien@ibmt.fraunhofer.de (L.W.); hagenvonbriesen@gmail.com (H.v.B.)

**Keywords:** blood–brain barrier (BBB), in vitro model system, human induced pluripotent stem cells (hiPSs), directed differentiation, brain capillary endothelial cells (BCECs)

## Abstract

In vitro model systems of the blood–brain barrier (BBB) play an essential role in pharmacological research, specifically during the development and preclinical evaluation of new drug candidates. Within the past decade, the trend in research and further development has moved away from models based on primary cells of animal origin towards differentiated models derived from human induced pluripotent stem cells (hiPSs). However, this logical progression towards human model systems from renewable cell sources opens up questions about the transferability of results generated in the primary cell models. In this study, we have evaluated both models with identical experimental parameters and achieved a directly comparable characterisation showing no significant differences in protein expression or permeability even though the achieved transendothelial electrical resistance (TEER) values showed significant differences. In the course of this investigation, we also determined a significant deviation of both model systems from the in vivo BBB circumstances, specifically concerning the presence or absence of serum proteins in the culture media. Thus, we have further evaluated both systems when confronted with an in vivo-like distribution of serum and found a notable improvement in the differential permeability of hydrophilic and lipophilic compounds in the hiPS-derived BBB model. We then transferred this model into a microfluidic setup while maintaining the differential serum distribution and evaluated the permeability coefficients, which showed good comparability with values in the literature. Therefore, we have developed a microfluidic hiPS-based BBB model with characteristics comparable to the established primary cell-based model.

## 1. Introduction

The blood–brain barrier (BBB) is comprised of brain capillary endothelial cells (BCECs) surrounded by a basement membrane with embedded pericytes and closely associated astrocytes [1,2]. Due to its highly selective nature when it comes to transport processes, model systems are in high demand in drug discovery and pharmacological research [3,4]. Within the last 10 years, BBB model systems have quickly evolved from models based on either primary cell sources [5,6,7,8] or immortalised cell lines of animal [5,9] or human origin [10,11] to model systems derived from differentiated human induced pluripotent stem cells (hiPS) [12,13,14]. The differentiation process of the reported hiPS-based model system has been improved and the model itself extensively characterised [15,16,17]. The use of co-culture models is also often discussed as a measure for improving barrier properties and correlating the models more closely with the in vivo situation [18,19]. However, the ability to accurately predict the in vivo situation remains questionable when evaluating the advantages and disadvantages of these models [19,20,21,22]. All values used for the comparative studies are derived from single publications. Di Marco et al. [23] have recently shown a good correlation with respect to permeability characteristics when comparing a hiPS-based BBB model with a porcine model system based on a library of 23 compounds. Both models used here were co-cultured with astrocytes, thus ensuring good comparability of the results. Over the years, a large number of studies and pharmacological evaluations have, though, been based on comparatively simple models, consisting only of endothelial cells as a barrier for the transport of substances [22]. Therefore, a direct comparison of the barrier properties of primary and hiPS-based BBB models consisting of endothelial cells, without co-culture with other cell types or their influence, will provide new insights into the reliability of such data.

**Figure 1:**
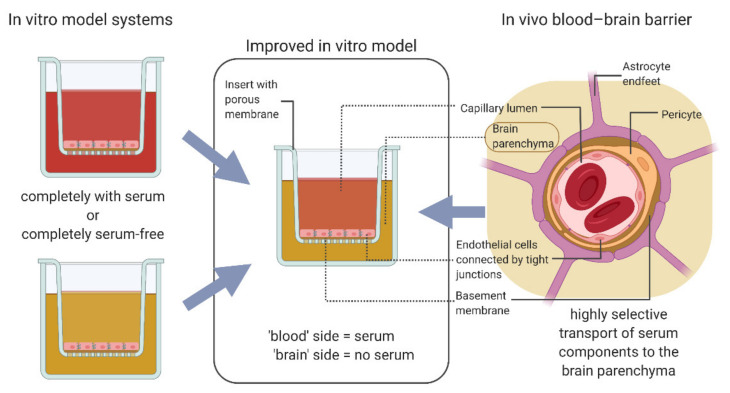
Closing the gap between in vitro models and in vivo state. In vitro models of the blood–brain barrier (BBB) are cultured with identical media on both sides of the cellular layer, either with serum or under completely serum-free conditions. In vivo, the BBB utilises highly selective transport mechanisms to regulate the nature and amount of serum components allowed to cross into the brain parenchyma. Applying this understanding to the culture conditions of an in vitro model system reveals a simple but important step towards more closely modelling this situation: medium containing serum is applied to the “blood” side of the cellular layer; medium without serum is applied to the “brain” side. Created with BioRender.com.

Additionally, though a recent study has shown a good correlation between in vivo and in vitro permeability in a co-culture model of human hiPS-BCECs and rat glial cells [24], one of the most basic BBB characteristics remains unanalysed. In the human brain in vivo, the blood–brain barrier functions as a highly selective membrane allowing only a very limited number of compounds and specific plasma proteins access to the brain parenchyma [25,26,27]. Therefore, the presence of complete serum as it is used in in vitro modelling should be restricted to one side of the cellular layer in a membrane model—the side representing the capillary interior (Figure 1). Established in vitro models either culture BCECs in completely serum-free conditions [28] or apply medium with a low serum content to both sides of the membrane carrying the cells [16,17,18,19,23]. We have investigated how in vitro blood–brain barrier models, one from primary cells and one hiPS-derived, reacted when serum was introduced to the medium only on the side of the membrane representing the capillary interior. We analysed whether this had an impact on improving the model systems and if this serum distribution could better represent the in vivo situation. The final investigated consideration was the influence of fluidic flow on the optimized blood–brain barrier model [29]: we inserted the blood–brain barrier on a membrane between two microfluidic channels and measured the permeability of relevant marker substances.

## 2. Materials and Methods

Cell culture media and solutions were purchased from Fisher Scientific (Schwerte, Germany) unless stated otherwise. The hiPS cell line UKKi011-A was obtained from the European Bank for induced pluripotent stem cells (EBiSC).

### 2.1. Isolation of Primary Porcine Brain Capillary Endothelial Cells (pBCECs)

**Figure 2:**
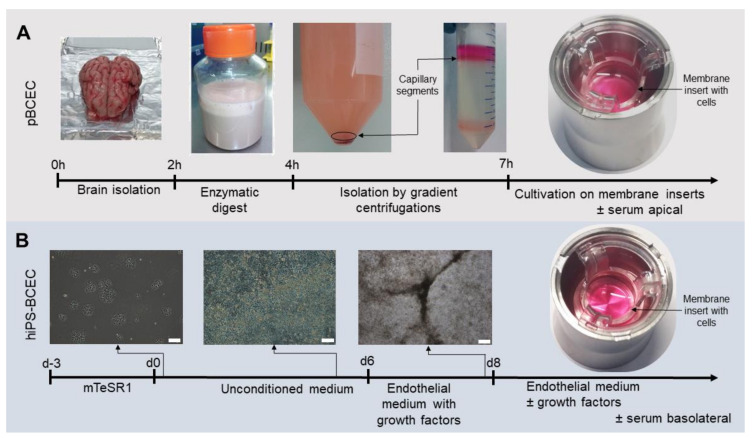
Generation of in vitro BBB models. (**A**) pBCECs were cultivated after isolation from a fresh brain of *Sus scrofa domestica* by consecutive enzymatic digestion and gradient centrifugation steps before seeding the isolated cells on membrane inserts to re-establish the BBB-specific cell–cell connections. (**B**) hiPS-BCECs were generated from hiPSs seeded to achieve a specific cell density at the start of the differentiation and cultivated for 6 days in unconditioned medium and two days in endothelial medium with growth factors, after which the cells were passaged and seeded on membrane inserts for cultivation of the specific BBB endothelial cells. Scale bar: 200 µm.

pBCECs were isolated from whole fresh porcine skulls of *Sus scrofa domestica* as described earlier [8] with some modifications (Figure 2A). In short, freshly slaughtered pig skulls were kindly provided by a local slaughterhouse in Zweibrücken, Germany. The animals were killed according to EG 1099/2009 of the Council of the European Commission on the protection of animals at the time of slaughter dated 24 September 2009. After opening and removing the skull cap, the brain was removed whole, the meninges were removed and the grey matter was separated from the white matter with a scalpel. The grey matter was collected and incubated with preparation medium (M199 medium with 1% penicillin/streptomycin, 1% gentamicin and 0.7 mM GlutaMAX) and dispase (Corning, Amsterdam, The Netherlands) at a final concentration of 3.1 U mL^−1^ for 2 h at 37 °C with stirring at 680 rpm. The dissolved brain tissue was layered on a discontinuous density gradient with a density of 1.0488 g cm^−3^ (Percoll (GE Healthcare, Solingen, Germany) and DMEM) in a ratio of 1 part liquefied tissue to 1 part gradient solution and centrifuged for 10 min at 2200× *g* and 4 °C without break during deceleration. From the resulting gradient, the capillary segments collected in a cloud near the bottom of the centrifuge tubes were collected in a new centrifuge tube, mechanically disrupted and mixed with an excess of preparation medium. The mixture was strained through a cell strainer with 100 µm pore size (Corning, Amsterdam, The Netherlands) and centrifuged again for 20 min at 230× *g* and 4 °C to collect the cells. The medium was removed until only 1 mL remained, mixed with 1 mL 466 U mL^−1^ collagenase (Biochrom, Berlin, Germany) and incubated at 37 °C and 150 rpm agitation for 30 min. After 27 min, 2 mL DNase I (Roche, Basel, Schweiz) was added for a final concentration of 2 × 10^−3^ U mL^−1^. The enzyme solutions were diluted with 7 parts preparation medium, and the solution was centrifuged for 10 min at 166× *g* and 4 °C. The supernatant was removed, the pellet resuspended in 1 mL preparation medium and carefully layered on a continuous density gradient and centrifuged for 20 min at 792× *g* and 4 °C without break during deceleration. The continuous density gradient was established by mixing Percoll (GE Healthcare, Solingen, Germany) and PBS to a final amount of 35 mL and a density of 1.065 g cm^−3^ followed by centrifugation for 45 min at 14,500× *g* and at room temperature without break during deceleration. The pBCECs were contained in the topmost pink band after centrifugation. The band was collected, diluted with preparation medium and centrifuged at 10 min at 166× *g* and 4 °C to collect the cells. The supernatant was removed and the pellet was resuspended in an appropriate volume of plating medium (preparation medium with 10% newborn calf serum) depending on pellet size. Cells were counted and plated at a density of 3.6 × 10^5^ cells cm^−2^ on Transwell^®^ filter membranes (Corning, Amsterdam, The Netherlands) with a pore size of 3.0 µm coated with 100 µg mL^−1^ rat tail collagen type I (Corning, Amsterdam, The Netherlands) in 0.02 N acetic acid. The cells were cultured in an incubator at 37 °C, 5% CO_2_, 90% relative humidity. After 1 h, the medium was replaced with new plating medium to remove unattached cells and after 24 h the medium was exchanged with culture medium (DMEM/F12 with 1% penicillin/streptomycin, 1% gentamicin, 0.75% GlutaMAX and 550 nM hydrocortisone (SigmaAldrich, Taufkirchen, Germany)). Experiments were conducted on day 4 or 5 after plating when the TEER value exceeded 300 Ω cm^2^. For experiments with serum in the apical compartment, 5% fetal bovine serum was added to the culture medium starting 24 h after plating the cells.

### 2.2. Differentiation of hiPSs to hiPS-BCECs

The stem cell line UKKi011-A was differentiated according to the protocol published by Stebbins et al. [30] with slight modifications (Figure 2B). Cells were seeded at a density of 5 × 10^3^ cells cm^−2^ on Matrigel-coated (Corning, Amsterdam, The Netherlands) 6-well plates and cultivated for 3 days in mTeSR1 medium (Stem Cell Technologies, Vancouver, BC, Canada) before initiating the differentiation. On day 0, the mTeSR1 medium was switched to unconditioned medium (DMEM/F12 with 20% knock-out serum replacement, 1% non-essential amino acids, 0.5% GlutaMAX and 0.1 µM 2-mercaptoethanol) and medium was exchanged daily for 5 days. On day 6, the medium was changed to endothelial medium with growth factors (heSFM with 1% platelet-poor plasma-derived human serum (PDS; SigmaAldrich, Taufkirchen, Germany), 20 ng mL^−1^ bFGF (R&D Systems, Minneapolis, MN, USA) and 10 µM retinoic acid (SigmaAldrich, Taufkirchen, Germany)). On day 8, cells were dissociated with Accutase for 25 min at 37 °C and seeded at a density of 1 × 10^6^ cells cm^−2^ in endothelial medium with growth factors. Cells were seeded onto membrane inserts with 3.0 µm pore size (VWR International, Darmstadt, Germany) and coated with 400 µg mL^−1^ human collagen IV (SigmaAldrich, Taufkirchen, Germany) and 100 µg mL^−1^ fibronectin (SigmaAldrich, Taufkirchen, Germany) in 0.05% acetic acid. The medium was changed 24 h after seeding to either endothelial medium without growth factors (heSFM with 1% PDS) or enriched endothelial medium (heSFM with 5% PDS) on the apical side and heSFM on the basolateral side. Experiments were performed on day 10 or 11.

### 2.3. Transendothelial Electrical Resistance Measurements

The transendothelial electrical resistance (TEER) and capacity of the cellular monolayers were automatically measured every hour via impedance spectroscopy, using the cellZscope device (Nanoanalytics, Münster, Germany). Therefore, the inserts were incubated within the cellZscope module in an incubator at 37 °C, 5% CO_2_ and 90% relative humidity from the time of seeding until the experiments were concluded. The module was connected to an external controller and computer. Media exchanges or sampling were performed between measurements. Data were exported to Excel for further analysis.

### 2.4. Microfluidic Setup

The microfluidic studies were performed using the commercial Quasi Vivo^®^ QV600 system (Kirkstall Ltd., York, UK). On day 8 of the differentiation of Ukki011-A cells to hiPS-BCECs, the cells were seeded on Millicell^®^ inserts (Merck KGaA, Darmstadt, Germany) at a density of 1 × 10^6^ cells mL^−1^ and cultivated as described above. On day 10, when the barrier has been established, the membrane containing the blood–brain barrier was inserted into the QV 600 chamber. The apical channel was filled with enriched endothelial medium (heSFM with 5% PDS), while heSFM was added to the basolateral channel. A fluidic flow with a flow rate of 200 µL min^−1^ was established and experiments were performed for up to 4 days.

### 2.5. Antibody Staining

For the analysis of protein expression, pBCECs and hiPS-BCECs were fixed with acetone for 2 min, washed twice with PBS and membranes were excised with a scalpel and transferred to well plates for staining. The membranes were blocked in PBS with 1% bovine serum albumin (BSA) at room temperature for 1 h. Primary antibodies for claudin-5 (abcam, Cambridge, UK), occludin and P-gp of Glut-1 (all obtained from Santa Cruz Biotechnology, Santa Cruz, CA, USA) were diluted in PBS with 1% BSA and incubated with the fixed cells at 4 °C overnight. Membranes were washed twice with PBS with 1% BSA and incubated with the secondary antibody rabbit anti-mouse Alexa Fluor^®^ 555 at room temperature for 1 h, washed twice with PBS and immobilised between two glass coverslips with VECTASHIELD^®^ HardSet™ Mounting Medium with DAPI (Vector Laboratories, Burlingame, CA, USA). After drying overnight, the samples were analysed with a Leica TCS SP8 confocal microscope (Leica Microsystems, Wetzlar, Germany).

### 2.6. Transport Studies

The permeability coefficient of sodium fluorescein (SigmaAldrich, Taufkirchen, Germany) was determined for pBCECs and hiPS-BCECs and those of [14C]-diazepam and [14C]-inulin (both from Hartmann Analytic GmbH, Braunschweig, Germany) only for hiPS-BCECs. The substances of interest were added to the apical side of the membranes, incubated for 3 h at 37 °C, 5% CO_2_ and 90% relative humidity, and then the samples were collected from the apical and basolateral compartment for measurement. In the microfluidic setup, sodium fluorescein and caffeine (SigmaAldrich, Taufkirchen, Germany) were added to the apical channel and samples were collected 4, 24, 48 (only caffeine) and 72 h after application from both fluidic channels. For comparison, samples in the static setting were collected at the same time points with a separate insert for each time point.

Sodium fluorescein was added to a final concentration of 1 µg mL^−1^, the samples were transferred to black 96-well plates and fluorescence was measured at an excitation of 485 nm and emission of 535 nm with a Tecan infinite 200 plate reader (Tecan Group Ltd., Männedorf, Switzerland). Standard dilutions of sample media were used for quantification and sample media from cell wells without sodium fluorescence application were used for background subtraction. All measurements were performed in duplex at least.

Per membrane insert, [14C]-diazepam and [14C]-inulin were added in a concentration of 13 kBq. After incubation, the apical and basolateral medium were collected separately, transferred to plastic vials containing 5 mL Ultima Gold scintillation liquid (Perkin Elmer, Waltham, MA, USA) and radioactive decay was measured with a Tri-Carb 2910TR Scintillation counter (Perkin Elmer, Waltham, MA, USA).

Caffeine was added to a final concentration of 100 µM to the apical circuit. Samples were collected from both the apical and basolateral circuits, filtered using Whatman Mini-UniPrep™ filters (VWR International, Damstadt, Germany) and caffeine concentrations measured using HPLC. Based on the method of Elberskirch et al. [31], the amount of caffeine was determined by an Agilent 1260 HPLC (Agilent Technologies, Santa Clara, CA, USA) using a Poroshell 120 EC-C18 column (2.1 × 100 mm, 2.7 µm; 695775-902, Agilent Technologies) connected to a guard column (Poroshell 120 EC-C18, 2.7 μm, 2.1 × 5 mm, Agilent Technologies). Separation was achieved with a gradient; the mobile phase consisted of three running agents: water (solvent A; sterile filtered 0.2 µm pore), acetonitrile (Solvent B; J.T. Baker) and 0.1% trifluoroacetic acid (TFA) in water (Solvent C; TFA; Fisher Scientific) (Table 1). At a flow rate of 0.4 mL min^−1^, 5 µL of sample was injected and separated at a column temperature of 60 °C. Detection was achieved using a Diode-Array-Detector (DAD) at a wavelength of 275 nm.

The permeability coefficients were calculated with the following Equation (1), assuming steady state flux:

(1) $$P_{\mathrm{app}}=\frac{c_{\mathrm{basolateral}}\;\times V_{\mathrm{basolateral}}}{t\times A\times c_{\mathrm{apical}}},$$

where P_app_ (cm s^−1^) is the permeability coefficient, c_basolateral_ and c_apical_ are the substance concentrations on the basolateral and apical side of the membrane, respectively (µg mL^−1^ or kBq mL^−1^), V_basolateral_ (mL) is the sample volume at the basolateral side, t (s) the incubation time and A (cm^3^) the area of the membrane [32].

### 2.7. Statistical Analysis

The data was expressed as mean ± standard deviation (S.D.). The statistical significance was determined with Origin software (Originlab, Northampton, MA, USA) using the Student’s *t*-test or two-way ANOVA analysis. 

## 3. Results

### 3.1. Comparison of Models According to Established Protocols

**Figure 3:**
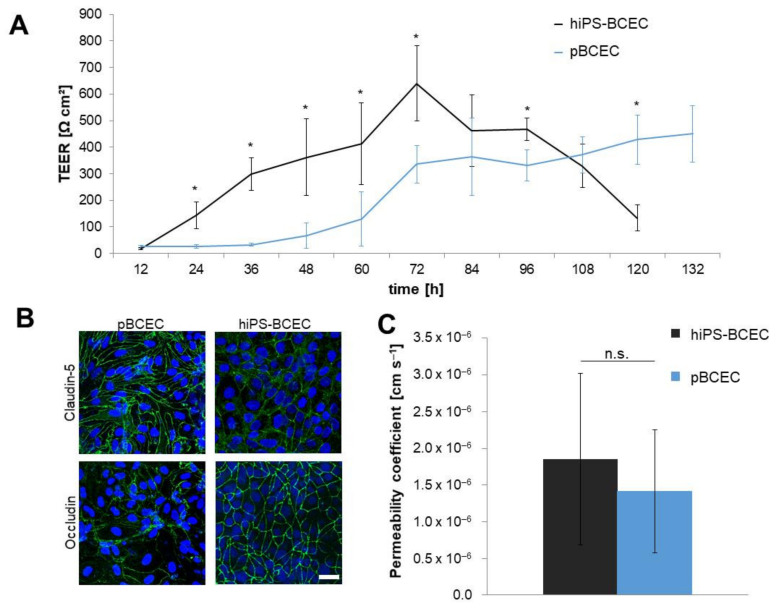
Barrier properties of pBCECs and hiPS-BCECs in in vitro models. (**A**) pBCECs were freshly isolated and seeded on collagen-coated Transwell^®^ inserts. hiPS-BCECs were differentiated for 8 days before dissociating and seeded on collagen- and fibronectin-coated membrane inserts. TEER values were measured from the time of seeding on the inserts with 3.0 µm pore size for both cell models. Mean ± S.D. was calculated with at least three biological replicates, * *p* < 0.05 for pBCECs compared to hiPS-BCECs. (**B**) Cells were fixed at the time of maximum TEER and stained for claudin-5 or occludin protein expression (green) and nucleus (blue). Scale bar: 25 µm. (**C**) Determination of permeability coefficients was performed at the time of maximum TEER value ± 12 h. To the apical compartment, 1 µM sodium fluorescein was applied and samples were taken for measurement after 3 h of incubation. Mean ± S.D. was calculated from at least three biological replicates; n.s. *p* > 0.05.

Primary BCECs were isolated from pig brains and cultivated as described above (Figure 2A). Maximum TEER values of 450 ± 105 Ω cm^2^ were achieved 132 h after seeding the isolated cells on membrane inserts (Figure 3A). hiPS-derived BCECs were differentiated according to Stebbins et al. [30] and adjusted for the specific cell line (Figure 2B). After 8 days of initial differentiation, the cells were seeded on membrane inserts and achieved a maximum TEER value of 640 ± 140 Ω cm^2^ approximately 72 h after seeding on the membranes before decreasing again. The maximum TEER value, therefore, is higher by approximately 190 Ω cm^2^ in hiPS-derived BCECs when compared to primary cell-derived ones. While confluence of the monolayer on the membrane inserts was achieved faster for hiPS-BCECs than for pBCECs (2 h compared to 12 h, data not shown), the hiPS-BCECs were also seeded at a higher density (hiPS-BCECs: 1 × 10^6^ cells cm^−2^; pBCECs: 3.6 × 10^5^ cells cm^−2^). In general, the time-dependent course of the TEER values differed significantly. The hiPS-BCECs reached their maximum TEER value after 72 h and achieved TEER values higher than 300 Ω cm^2^ as soon as 36 h after being seeded on the membranes, whereas the pBCECs achieved values such as these after 72 h at the earliest. The TEER development of the pBCECs was slower and they only had higher values than the hiPS-BCECs in direct comparison after more than 108 h of culture on the inserts. At this point, the TEER values of the hiPS-BCECs had already started to decrease again.

Immunofluorescence staining showed similar expression of specific BBB tight junction proteins in both model systems: claudin-5 and occludin (Figure 3B). The proteins were correctly located between neighbouring cells at the cell–cell border. The cell shapes showed clear differences in both systems. While the pBCECs showed a typical elongated appearance, the hiPS-BCECs more closely resembled a cobblestone in their shape.

Protein expression and TEER values indicated reliable models, but for a final evaluation a more functional marker was also investigated: the permeability coefficient of sodium fluorescein as a model substance (Figure 3C). The measured values of 1.85 × 10^−6^ and 1.41 × 10^−6^ cm s^−1^ for pBCECs and hiPS-BCECs, respectively, were both higher than 1 × 10^−6^ cm s^−1^. Permeability values of 1 × 10^−6^ cm s^−1^ or less have been discussed as representing an intact BBB [30,33]. Therefore, these comparatively simple models cannot quite achieve in vivo-like properties but are still a viable tool to study the BBB.

### 3.2. Protocol Improvement for More In Vivo-like Characteristics

Comparing the cultivation protocols specifically for the phase on the membrane inserts, a small but significant difference becomes obvious: the pBCEC model completely abolished the use of serum in this phase, while the hiPC-BCECs were cultivated in the presence of 1% serum during the whole membrane cultivation phase. For the in vivo BBB, serum and more specifically serum proteins were largely restricted to the blood side of the BBB when not transferred across by specific transporters. To mimic this situation more closely, the media in both models were adjusted with the first regular medium exchange after seeding on the inserts. The apical side in these model systems represented the blood side, while the basolateral side modelled the brain environment. The pBCECs received a medium with the addition of 5% FCS on the apical side and still without serum on the basolateral side. The hiPS-BCECs were adjusted similarly by increasing the amount of platelet-poor plasma-derived human serum (PDS) in the medium for the apical side to 5% while simultaneously completely removing serum from the medium for the basolateral side.

**Figure 4:**
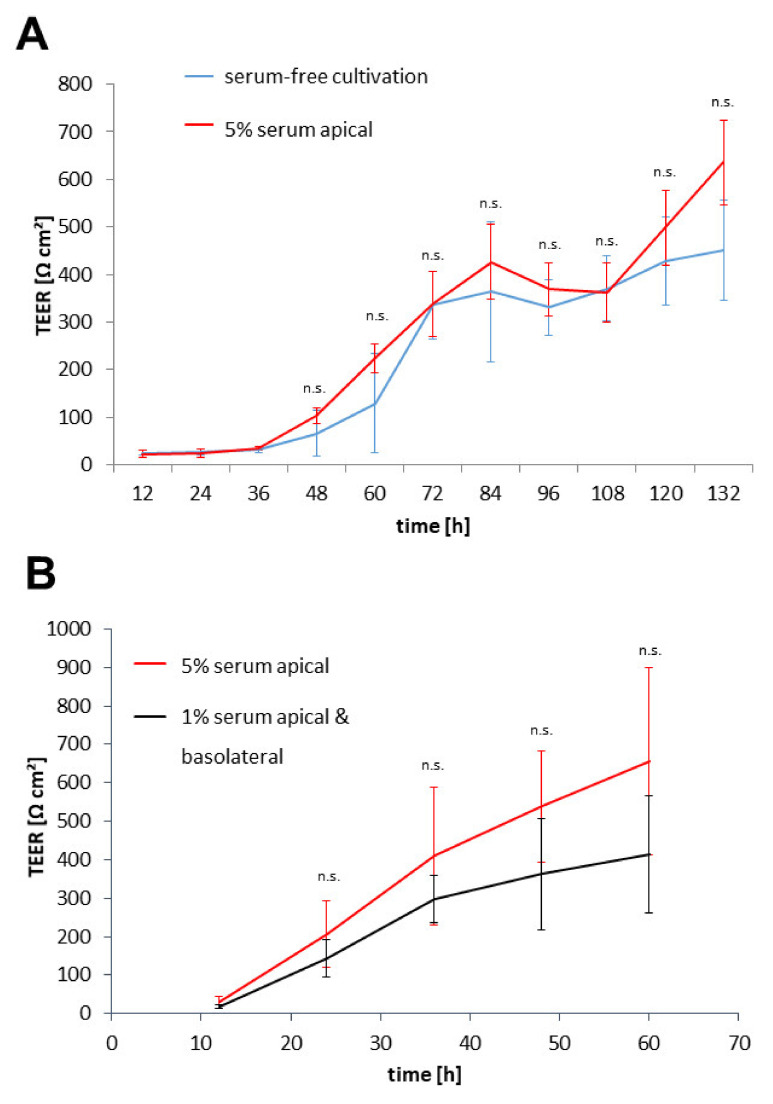
TEER development in reaction to a change in serum contents in the media. (**A**) pBCECs were seeded on collagen-coated Transwell^®^ inserts with 3.0 µm pore size after isolation. TEER values were measured continuously. The medium was exchanged 24 h after seeding with either culture medium (serum-free, blue line) or culture medium with 5% FCS on the apical side and serum-free culture medium on the basolateral side (red line). Data shows mean ± S.D. at 12 h intervals for at least three biological replicates. (**B**) After 8 days of differentiation, hiPS-BCECs were seeded on collagen IV–fibronectin-coated membrane inserts with 3.0 µm pore size and TEER was measured continuously. The medium was exchanged 24 h after seeding with either endothelial medium without growth factors (1% PDS, black line) or with enriched endothelial medium (5% PDS) on the apical side and endothelial medium without serum on the basolateral side (red line). Data shows mean ± S.D. at 12 h intervals for at least three biological replicates; n.s. *p* > 0.05.

Analysing the change in TEER values showed an increase in maximum TEER values in both the primary and the hiPS-derived model system. For the pBCECs, TEER increased from 450 ± 105 Ω cm^2^ to 635 ± 90 Ω cm^2^ (Figure 4A), and for the hiPS-BCECs, an increase at 60 h after seeding to 656 ± 243 Ω cm^2^ from 413 ± 155 Ω cm^2^ was measured (Figure 4B). For both cell models, the increase in TEER was accelerated. While there was a slight drop in TEER values in the pBCEC model between 84 h and 96 h, the TEER values started increasing again after another medium exchange. There was no such notable drop in TEER values for the hiPS-BCEC model. The TEER development indicated that the barrier itself was capable of withstanding the osmotic pressure introduced to the barrier during the medium exchanges with different serum contents on the opposing sides of the barrier.

**Figure 5:**
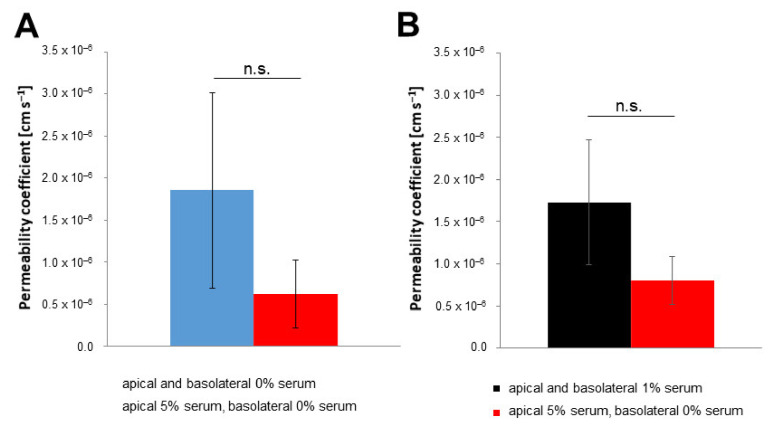
Change in permeability of sodium fluorescein dependent on media serum profiles. Determination of permeability coefficients was performed at the time of maximum TEER value ± 12 h for (**A**) pBCECs and (**B**) hiPS-BCECs. To the apical compartments of the models with standard media (pBCECs—blue; hiPS-BCECs—black) or medium with 5% serum on the apical side and serum-free medium on the basolateral side (both—red), 1 µM sodium fluorescein was applied. Samples were taken for measurement after 3 h of incubation. Mean ± S.D. was calculated for at least three biological replicates; n.s. *p* > 0.05.

This increase in TEER values was also reflected in the permeability coefficients. The permeability of sodium fluorescein in both model systems was lowered to values smaller than 1 × 10^−6^ cm s^−1^ by changing the serum profiles of the media (Figure 5). The permeability coefficients with different media serum levels dropped to 6.2 × 10^−7^ cm s^−1^ and 8.0 × 10^−7^ cm s^−1^ for pBCECs and hiPS-BCECs, respectively. This lowering of permeability coefficients in combination with the steady increase in TEER values also indicated that the barrier was not disrupted by medium exchange. In contrast, it was positively impacted by differing serum concentrations on the opposing sides of the cell layer.

### 3.3. Characterisation of In Vivo-like hiPS-Based Model

**Figure 6:**
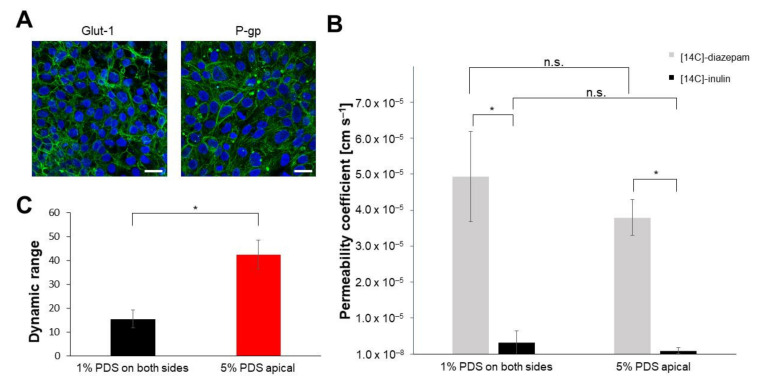
Additional characterisation of the hiPS-BCEC in vitro model. (**A**) Cells were fixed at the time of maximum TEER and stained for Glut-1 or P-gp expression (green) and nucleus (blue). Scale bar: 25 µm. (**B**) Determination of permeability coefficients was performed at the time of maximum TEER value ± 12 h. To the apical side, 13 kBq [14C]-diazepam or [14C]-inulin were applied, followed by incubation for 3 h, after which samples were taken from both the apical and basolateral sides for measurement. Mean ± S.D. was calculated for at least three biological replicates; * *p* < 0.05, n.s. *p* > 0.05. (**C**) The dynamic range as a quantification of selective barrier permeability was determined by dividing the permeability coefficient of diazepam by the permeability coefficient of inulin. Mean ± S.D. was calculated for at least three biological replicates; * *p* < 0.05.

The model system most closely resembling the in vivo situation of humans, the hiPS-BCEC model with media with different serum contents, was further characterised to investigate how accurate the model is. To this end, the expression of the BBB-specific transport proteins P-gp and Glut-1 was analysed (Figure 6A).

Both transporters were strongly expressed in this model system. They were localised in close association with the cell–cell boundaries and, in the case of P-gp, also in a more diffuse distribution in the cytoplasm as well as in some vesicle-like structures.

Finally, the transport of substances with a well-known transport behaviour in vivo was measured. The lipophilic compound diazepam was selected as an example of a compound capable of crossing the BBB, while inulin was selected as a hydrophilic substance and known paracellular transport marker. Both compounds rely on passive transport across the BBB [34]. To ensure accurate measurements, the substances were used in a radiolabelled format: [14C]-diazepam and [14C]-inulin. Scintillation counting of apical and basolateral samples after 3 h incubation time revealed differences in the permeability coefficients of both substances (Figure 6B). The permeability coefficients of [14C]-diazepam and [14C]-inulin with equal serum distributions were 49.3 × 10^−6^ and 3.19 × 10^−6^ cm s^−1^, respectively, while they dropped to 37.9 × 10^−6^ cm s^−1^ for [14C]-diazepam and 0.89 × 10^−6^ cm s^−1^ for [14C]-inulin when serum was only present in the apical medium. The dynamic range was calculated by dividing the permeability value determined for a lipophilic compound by the permeability value of a hydrophilic compound [35]. This range can illustrate the capability of cells to form tight junctions and thus to restrict paracellular transport of hydrophilic compounds by putting the potentially restricted paracellular transport of hydrophilic compounds in a relation to the not impacted transcellular transport of lipophilic compounds. The dynamic range between both permeability values (lipophilic diazepam and hydrophilic inulin) increased significantly from 15.5 ± 3.9 to 42.5 ± 6.0 (Figure 6C), indicating a tight barrier capable of clearly differentiating between compounds with expected high or low permeability. The permeability coefficients, as well as the dynamic range, were improved by switching from media with low serum content on both sides of the barrier to serum-containing medium at the apical side and serum-free medium at the basolateral side.

### 3.4. Influence of Fluidic Flow on Permeability

**Figure 7:**
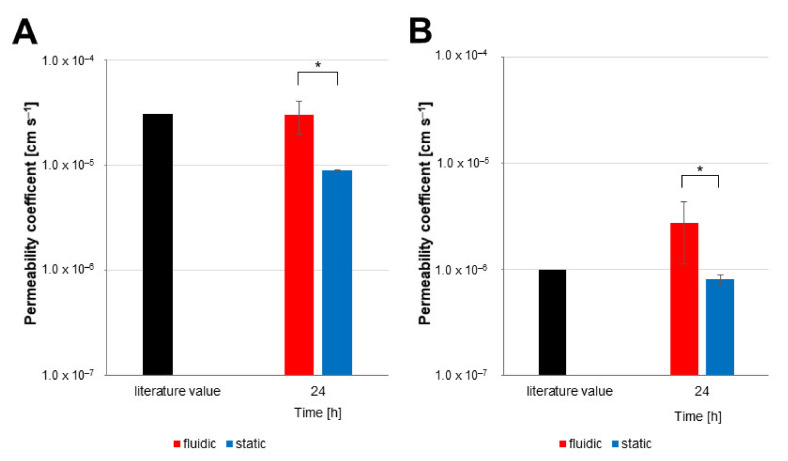
Permeability coefficients of caffeine and sodium fluorescein in the presence or absence of fluidic flow. Determination of permeability coefficients was performed for (**A**) caffeine and (**B**) sodium fluorescein. To the apical compartment of the models, 100 µM caffeine and 1 µM sodium fluorescein was applied at day 10 of development (fluidic—red; static—blue) and samples of both circuits were taken for measurement at the indicated time point. Mean ± S.D. was calculated for three biological replicates, * *p* < 0.05.

To evaluate the effect of fluidic flow as found in the brain capillaries in vivo, the hiPS-BCECs cultivated with different serum contents in their media on opposite sides of the barrier were inserted into a commercially available microfluidic chamber with two separate fluidic channels. The permeability values for sodium fluorescein, as a compound with low permeability, and caffeine, as a compound with high permeability, were determined and compared with values in the literature (Figure 7).

As can be seen in Figure 7A, the permeability coefficient of caffeine in the fluidic model was 3.04 × 10^–5^ ± 1.04 × 10^–5^ cm s^−1^ and was therefore very close to the literature value of 3.1 × 10^–5^ cm s^−1^ [24]. In contrast, the permeability coefficient in the static system differed significantly and was lower than the literature value at 0.90 × 10^–5^ ± 1.22 × 10^–7^ cm s^−1^.

**Figure 8:**
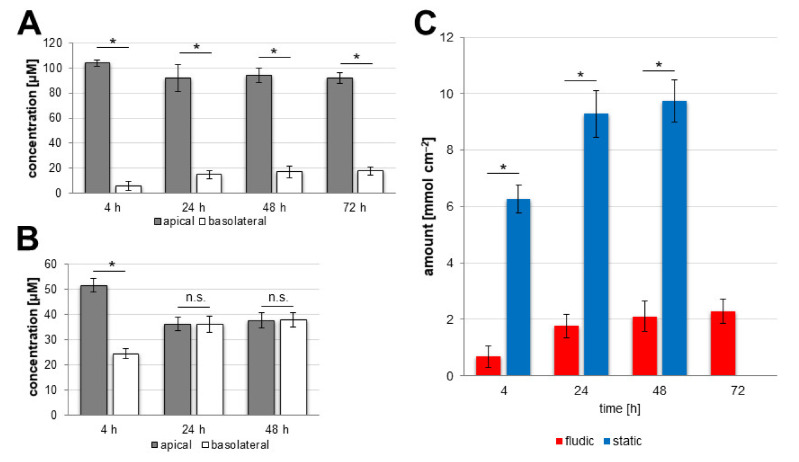
Transport of caffeine across the BBB in fluidic and static experimental settings. Determination of permeated caffeine was performed at different time points. To the apical compartment of the models, 100 µM caffeine was applied at day 10 of development and samples of both circuits were taken for measurement at the indicated time points. Distribution of caffeine between apical and basolateral compartment at different time points in the (**A**) fluidic and (**B**) static system. (**C**) Transported amount of caffeine dependent on BBB area for the fluidic (red) and static (blue) systems. Mean ± S.D. was calculated for three biological replicates; * *p* < 0.05, n.s. *p* > 0.05.

The permeability coefficients of sodium fluorescein (Figure 7B) showed higher values in the fluidic system than in the static model. The permeability value in the fluidic system was 2.75 × 10^–6^ ± 1.61 × 10^–6^ cm s^−1^. Only values below the literature value of 1 × 10^–6^ cm s^−1^ were regarded as representing a tight blood–brain barrier [30]. The permeability value in the static system was below this literature value at 0.81 × 10^–6^ ± 0.82 × 10^–7^ cm s^−1^. To better evaluate the differences observed for the permeability values for both systems, the caffeine permeability was investigated in more detail (Figure 8).

As can be seen in Figure 8A, the caffeine concentration in the basolateral side of the fluidic system increased with increasing experimental time from 5.91 ± 3.36 µM at 4 h until 17.72 ± 3.34 µM at 72 h. However, the increase in concentration slowed down after 24 h (14.85 ± 3.50 µM), increasing to 17.08 ± 4.44 µM at 48 h and then only minimally again until 72 h. The decrease in concentration in the apical side showed the same trend. In the fluidic system (Figure 8B), the situation was different. There, 24.46 ± 1.90 µM caffeine was measured on the basolateral side after 4 h. The values at 24 h and 48 h of 36.19 ± 3.18 µM and 37.97 ± 2.88 µM, respectively, were very similar to each other as well as to the values measured on the apical side at these time points: 36.30 ± 2.71 µM (24 h) and 37.74 ± 3.02 µM (48 h). This shows that the caffeine distribution reached an equilibrium between these time points. 

Due to the setups of both systems, the relation between BBB area and the amount of medium on the different sides of the BBB is very different. While these considerations were taken into account when calculating the permeability values, it was also interesting to directly compare the amount of transported caffeine when put into the context of the BBB area available for transport. As can be seen in Figure 8C, the amounts of caffeine transported at the different time points in both systems differed greatly. While 6.27 ± 0.49 mmol cm^−2^ caffeine was transported in the static system at 4 h, only 0.68 ± 0.39 mmol cm^−2^ was transported in the fluidic system. Due to the approached equilibrium, the values in the static system increased to 9.28 ± 0.92 mmol cm^−2^ and 9.74 ± 0.74 mmol cm^−2^ at 24 h and 48 h, respectively. In the fluidic system, the slowed increase shown in Figure 8A was also found here, with the values 1.77 ± 0.42 mmol cm^−2^, 2.11 ± 0.55 mmol cm^−2^ and 2.27 ± 0.43 mmol cm^−2^ at 24 h, 48 h and 72 h, respectively, but, in contrast to the static system, an equilibrium in the distribution of caffeine was not approached in this system. 

## 4. Discussion

In this study, we could show that the published protocols for pBCECs and hiPS-BCECs produce blood–brain barrier models with comparable characteristics in terms of TEER, sodium fluorescein permeability and tight junction protein expression. While the TEER values of the pBCECs were a mean of ~200 Ω cm^2^ lower than those of the hiPS-BCECs, no significant difference was found for the expression of the tight junction proteins claudin-5 and occludin nor for the permeability of sodium fluorescein. The decrease in TEER values observed in the hiPS-BCEC model after more than 84 h of culture was most likely based on a loss of specific cellular characteristics due to missing extracellular signals, as has been shown by Lippmann et al. [16]. No co-culture with other cells types found in the neurovascular unit [36], such as astrocytes [23] or pericytes, was necessary to produce good, reliable model systems, but this might have restricted the experimental window in the hiPS-BCEC model. However, a restriction to an experimental window of 48 h in pharmacological transport studies still provides many opportunities for study. This indicates that both model systems, even in this configuration restricted to a single cell type, have a comparable validity. Therefore, the selection of the model system can be based on other considerations, such as the specific underlying scientific question, the target organism being modelled or reasons that are even more mundane, for instance, the availability of either primary tissue or hiPS cells for differentiation. When considering a model system for pharmacological applications and transport studies with potential drug candidates for human medical treatment, the use of hiPS cells as source material is preferable to eliminate species differences that make the transferability of preclinical results to clinical trials challenging. The hiPS origin can even allow for the development of disease-specific model systems in cases where BBB permeability might be impacted by the underlying disease.

The maximum TEER values achieved in this study for the previously described cultivation protocols of 450 ± 105 Ω cm^2^ (pBCECs) and 640 ± 140 Ω cm^2^ (hiPS-BCECs) are notably lower than those reported by other groups [14,16,19,23,30]. In contrast, the permeability coefficients are comparable [19,30,37], indicating that the produced barrier models had a comparable functional tightness, even if the electrical resistance measurements provided lower values. The reason for the lower TEER values can be found when considering the membrane inserts used in this study. We only used membranes with a pore size of 3.0 µm, while the standard membrane pore size for other groups was 0.4 µm [16,17,19,23,30]. We have found that this larger pore size leads to a two- to three-fold decrease in TEER values (Supplementary Materials Figure S1). Taking this factor into account, the TEER values are within a comparable range to other published data. This also supports the frequently reported opinion that TEER values alone are not sufficient to evaluate the quality of an in vitro model system of the BBB [19,21,22]. Other parameters, such as permeability values and protein expression, have to be considered as well. As has been shown by Gaillard and Boer [38], the relationship between permeability and TEER values is not linear, suggesting that the influence of the TEER value above a certain threshold is largely irrelevant to a model’s behaviour as it relates to transport of substances. The evaluation of the quality of a BBB model, therefore, should not be based on the achieved TEER values but should include a number of other characteristics, especially the transport of model substances with known transport behaviour.

Our observation of the different serum concentrations in the BBB media and the subsequent attempt to change the serum concentrations in the apical and basolateral media of both model systems had a positive effect for primary and hiPS-derived cells. The TEER values under differing serum concentrations for the two sides of the BBB model increased in the pBCEC model by approximately 185 Ω cm^2^ and in the hiPS-BCEC model by ~243 Ω cm^2^. This increase in TEER correlates with a decrease in the sodium fluorescein permeability to values below 1 × 10^−6^ cm s^−1^ in both in vitro models. While the effects are not always significant, in our opinion it is always a desirable trait for any model system to mimic the in vivo situation as closely as possible. For the blood–brain barrier, this includes a strong restriction and selectivity when it comes to serum proteins found on the brain side of the barrier [39,40]. By only providing serum proteins on the side mimicking the capillary interior, the endothelial cells of the model are solely responsible for the amount and type of proteins transported across the barrier. This could also encourage the endothelial cells towards correct polarisation of their membrane-associated receptors and transport proteins, as is found in vivo [41,42,43]. The serum-free conditions on the basolateral side also open up new avenues for co-culture studies with neuronal cells, as they often prefer serum-free conditions with defined growth factors [44,45,46], without making it necessary to redesign the medium composition on the apical side that is not in direct contact with the co-cultured cells.

The investigation of the contrasting permeability of lipophilic and hydrophilic model substances in the form of diazepam and inulin in the hiPS-BCEC model system also revealed a notable change in the dynamic range when changing to different serum contents. While the decrease in the permeability coefficients for [14C]-diazepam and [14C]-inulin was not significant, the dynamic range more than doubled. This indicates that this BBB model with different serum contents on its sides is much better able to depict the important differences in permeability for lipophilic and hydrophilic compounds. While it is necessary to note that the permeability of diazepam, while passively transported across the BBB, is also largely reliant on the blood flow at the BBB [34,47], this is not a concern in this experimental setup. Here, the models are purely static; thus, the influence of flow conditions on diazepam uptake can be disregarded when evaluating the permeability values. 

The transport of lipophilic substances across the BBB can occur without direct involvement of receptors or transporters. In contrast, the transport of hydrophilic substances is completely dependent on these processes. Due to the uniform presence of tight junctions, paracellular diffusion of hydrophilic compounds is impossible for all but the smallest molecules. An increase in the dynamic range, the ability to differentiate between lipophilic and hydrophilic compounds concerning their transport rates, therefore indicates an increase in specificity, intact tight junction establishment and dependence on active transport processes. The higher the dynamic range, the better the model correlates with the in vivo situation.

Introducing the hiPS-BCEC model optimized with different serum contents on both sides of the barrier into a microfluidic channel led to surprising results when considering the investigated permeability values. It has been reported repeatedly that the addition of fluidic flow or shear stress will lead to an increase in TEER values and thus reduce the permeability of marker substances [48,49]. However, in our experiment the permeability coefficients for the marker substance sodium fluorescein were a little above the expected maximum permeability values in the fluidic setup. In contrast, the permeability coefficients for caffeine, a substance that is transported across the blood–brain barrier, were within the range of the values reported in the literature for an experimental window of up to 48 h, while the values started approaching an equilibrium after only 24 h in the static system. This strongly suggests that the fluidic system provides reliable results for actively transported substances over a longer experimental period. This distinctly extended experimental window also provides other options to evaluate the transport of pharmacological substances when applied together with other compounds, either in a simultaneous or a staggered manner. Interactions of different pharmaceutics can be investigated regarding their combined transport behaviour and effect on the BBB, including co-transport or transport inhibitions. Looking into the detailed transport behaviour, it is shown clearly that the amount of transported caffeine is lower in the fluidic system than in the static model, showing a correlation with the situation observed in other publications [50,51,52]. Especially in pharmacological studies, the fluidic setup with its large medium compartments on the different sides of the BBB allows for extended investigations, promising reliable results without the risk of the values being unreliable due to the establishment of a concentration equilibrium of the substances under investigation.

## 5. Conclusions

The direct experimental comparison of the primary cell-based pBCEC model and the hiPS-differentiated hiPS-BCEC model revealed no significant differences between the two models. The slight improvements in TEER and permeability of the hiPS-BCEC model have to be weighed against the intended scientific or pharmacological question in the context of model selection. Nonetheless, both models can be improved by the simple measure of applying media with different serum contents on the apical and basolateral sides of the model. This not only more closely resembles the situation in vivo but also improves the specific transport behaviour based on compound characteristics and relative solubility, therefore presenting a better model for pharmacological evaluation of drug candidates. Further changes to the established cellular model in terms of fluidic modelling have the potential to extend the experimental time frame, increasing the range of investigative options and sampling opportunities due to the larger quantities of medium utilised.

## Figures and Tables

**Table 1:** HPLC gradient according to Elberskirch et al. [31]. Solvent A: water; solvent B: acetonitrile; solvent C: 0.1% trifluoroacetic acid in water.

Time (min)	Solvent A (%)	Solvent B (%)	Solvent C (%)
0	76.5	8.5	15
0.5	76.5	8.5	15
2.5	42	38	20
4	0	57.5	42.5
10	0	57.5	42.5
14	76.5	8.5	15

## Data Availability

The data presented in this study are available on request from the corresponding author.

## Supplementary Materials

The following supporting information can be downloaded at: https://www.mdpi.com/article/10.3390/pharmaceutics14040737/s1, Figure S1: TEER development in reaction to membrane pore size.
