# Supplementary material for: Experimental Comparison of Primary and hiPS-Based In Vitro Blood–Brain Barrier Models for Pharmacological Research
Source: Pharmaceutics. 2022 Mar 29;14(4):737. doi: 10.3390/pharmaceutics14040737 (PMC9031459; doi:10.3390/pharmaceutics14040737)
Supplement: Supplementary file 1 [file pharmaceutics-14-00737-s001.zip › pharmaceutics-1660162-supplementary.pdf]

pharmaceutics

Supplementary Materials: Experimental Comparison of Primary and hiPS-Based In Vitro Blood–Brain Barrier Models for Pharmacological Research

Karin Danz, Tara Höcherl, Sascha Lars Wien, Lena Wien, Hagen von Briesen and Sylvia Wagner

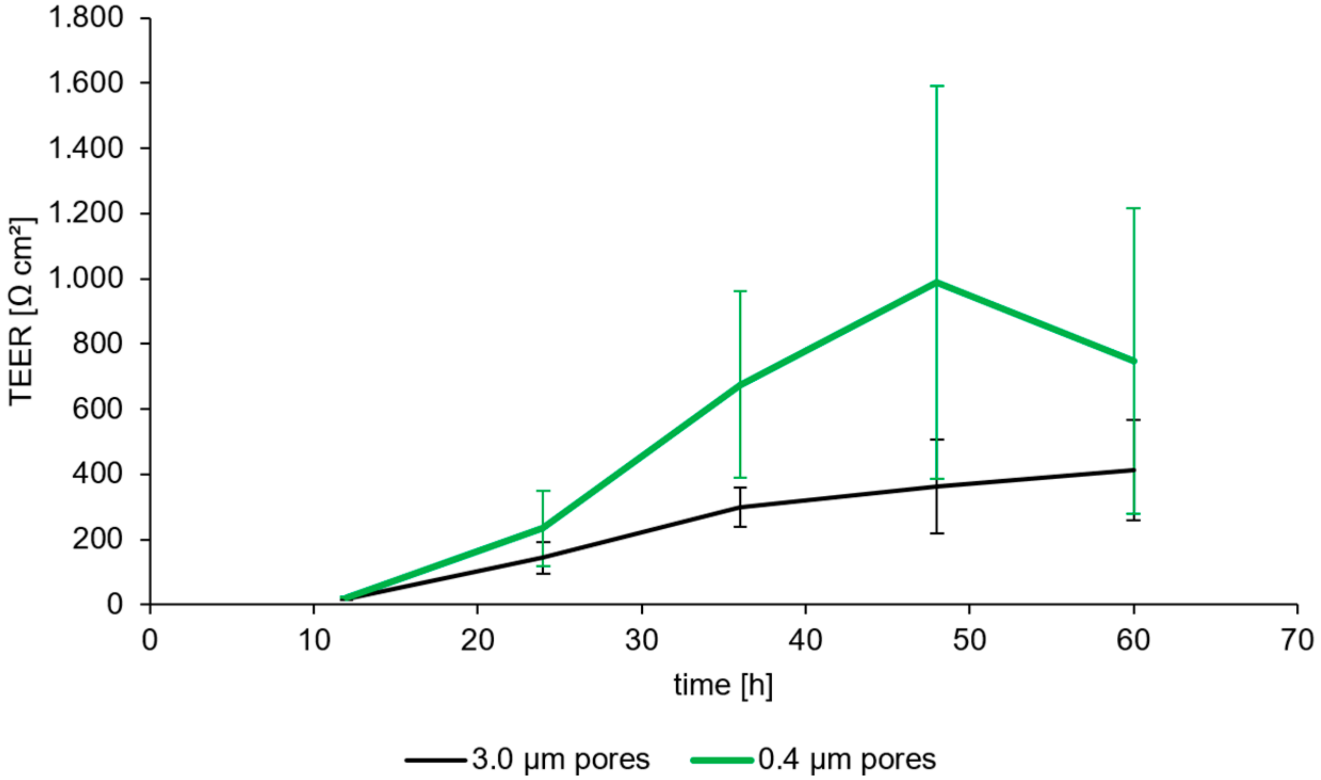

Figure S1. TEER development in reaction to membrane pore size. After 8 days of differentiation hiPS-BCEC were seeded on collagen IV-fibronectin-coated membrane inserts with 3.0 µm pore size (black line) or 0.4 µm pore size (green line) and TEER measured continuously. Data shows the mean ± S.D. in 12 h-intervals from at least three biological replicates.
